# Case Report: HSV-2 Encephalitis Presenting With Chorea; Effects of Infection Alone or Combination of Infection and Autoimmunity?

**DOI:** 10.3389/fneur.2021.790514

**Published:** 2021-12-10

**Authors:** Michael Kolesnik, Ahmad A. Ballout, Natasha Hameed, Souhel Najjar

**Affiliations:** Department of Neurology, Donald and Barbara Zucker School of Medicine at Hofstra/Northwell, New York, NY, United States

**Keywords:** herpes simplex 2 encephalitis, chorea, CSF negative, MRI negative, antibody negative

## Abstract

**Background:** Chorea as a symptom of late-onset post-infectious autoimmune encephalitis has been reported with HSV-1 but not HSV-2 encephalitis. Extrapyramidal symptoms are typically associated with the presence of anti-NMDA receptor antibodies but may also exist in antibody-negative individuals.

**Case:** This case highlights a patient who presented with mental status changes and chorea as the initial manifestation of HSV-2 encephalitis. The choreiform movements failed to respond to antiviral medications but were rapidly responsive to plasmapheresis, which, together with abnormal intrathecal immunoglobulin synthesis, suggests a potential contribution of parainfectious immune-mediated process. The patient made a full recovery and a complete resolution of the chorea.

**Discussion:** This is the first case associating HSV-2 encephalitis presentation with chorea. The neurological complications, including chorea, are largely related to active CNS HSV-2 infection, possibly together with triggered CNS autoimmunity despite undetectable CSF neuronal autoantibodies and normal neuroimaging. Early diagnosis and treatment with antiviral agent and immune therapies might be pivotal to optimize the clinical outcome.

## Introduction

Herpes Simplex Virus (HSV) is the most common viral cause of encephalitis (1). Given the limbic system predilection, it often manifests clinically with behavior changes, memory impairment, and language dysfunction. Extrapyramidal symptoms are less common. While this is most frequently caused by HSV-1, it can rarely be due to HSV-2 invasion in up to 2–10% of HSV encephalitis cases (2, 3).

HSV-2 may present as a primary infection or latent reactivation. Neurological complications related to primary infection are most commonly observed in neonates, such as the neonatal herpes simplex encephalitis (4). In contrast, primary HSV-2 infections in immunocompetent adults are often asymptomatic as the virus lays dormant in the sacral and trigeminal ganglia, which are sites for potential reactivation. Thus, neurological complications of HSV-2 infections in adults are often due to latent viral reactivation and include adult aseptic meningitis, recurrent aseptic meningitis, adult encephalitis and meningoencephalitis, rhombencephalitis, myelitis, radiculopathy, and cranial neuropathy (4, 5).

Autoimmune encephalitis is an immune mediated central nervous system (CNS) inflammatory process that is largely related to neuronal autoantibodies (6). Viruses, including HSV-1, can serve as immunological triggers, causing post-infectious autoimmune encephalitis in addition to meningoencephalitis (6–8). Early recognition and treatment of HSV meningoencephalitis and its post-infectious sequelae are important as it has been shown to reduce mortality from 70 to 16% (9, 10).

Chorea, among other movement disorders, as a sequela or relapse of HSV-1 encephalitis, is well documented (9–11). It is mechanistically linked to secondary post-infectious autoimmunity against neuronal surface proteins or receptors, such as N-methyl-D-aspartate receptor (NMDAR) and dopamine-2 receptor (12), although in some cases neuronal autoantibodies are not detected (6–11). Further, relapses associated with chorea are shown to be associated with a worse prognosis and a greater risk of long-term neurological deficits (12).

We report the first case of HSV-2 encephalitis presenting with chorea, in addition to acute mental status changes, presumably due to infection with, potentially, concurrent central nervous system (CNS) autoimmunity despite undetectable neuronal autoantibodies.

## Case Presentation

A 72-year-old woman with a history of gastritis and urinary incontinence presented with depressed level of consciousness after being found at her home. This is preceded by one day of confusion and lethargy as noted by her family. General examination and vitals revealed a fever of 38.6°C without meningismus. Neurological examination demonstrated a patient who was awake and oriented to self and location with noted psychomotor slowing in response to questioning and commands with noted impaired attention and distractibility during history taking. Cranial nerve testing was intact. The patient had full strength throughout her body and had clearly visible choreiform movements involving her neck and left upper extremity which were predominantly in the proximal part of the extremity. She was unable to suppress movements and was unaware of them when asked to control them. Reflexes were normal. Gait was not tested as it was unsafe to do so. Serologies demonstrated a normal white blood cell count and erythrocyte sedimentation rate with mildly elevated C-reactive protein of 6.8 mg/L (reference < 4.9). The patient was found to have a urinalysis that was positive for leukocyte esterase, bacteria, and white blood cells. She was empirically treated with ceftriaxone 1 g daily. However, despite several days of antibiotic coverage and urine cultures growing ceftriaxone-sensitive E. coli, the patient continued to have fevers, impaired attention and awareness, and chorea. It was decided to continue to search for a source of infection and hence, a lumbar puncture was performed. Cerebrospinal fluid (CSF) analysis revealed a pleocytosis of 234 cells/ul (reference 0–5 cells/ul) with 95% lymphocytic predominance, elevated protein of 72 mg/dl (reference 15–45 mg/dl), normal glucose of 49 mg/dl (reference 40–70 mg/dl), 15 present oligoclonal bands with CSF restriction, and increased IgG index of 2.2 (reference <0.7) with increased IgG synthesis of 32.3 mg/day (reference ≤ 8.0). CSF PCR was positive for herpes simplex virus 2. The remaining CSF infectious workup was negative, including HSV-1, VDRL, Lyme, cryptococcal antigen, bacterial and fungal cultures, and acid-fast stain. Urinalysis did not reveal any signs of infection. Blood cultures, HIV, Treponema antibody, and COVID-19 PCR were negative. A Mayo Clinic CSF autoimmune encephalitis panel was negative (Mayo Laboratories ID:ENC2). Serum autoimmune encephalitis panel was inadvertently omitted. MR brain with Gadolinium was unremarkable. The patient was started on acyclovir given the positive HSV-2 PCR without notable improvement in mental status or choreiform movements. On hospital day six, a parainfectious CNS autoimmunity was suspected for which the patient was started on intravenous immunoglobulin (IVIG), which was discontinued after two sessions due to severe hypotension. Alternatively, plasmapharesis was initiated on hospital day 9. It resulted in significant improvement of chorea which was noted after only one session and its complete resolution after the fourth session. Overall, she received a total of five plasmapharesis sessions and three weeks of acyclovir treatment. The patient's mental status, however, improved only minimally at the time of discharge to acute rehabilitation on hospital day 27.

### Outcome and Follow up

The patient successfully completed rehabilitation and returned to the neurology clinic three months following discharge. She achieved complete recovery without any neurological sequelae.

## Discussion

To the best of our knowledge, this is first case that associates HSV-2 encephalitis with chorea. Although this patient is immunocompetent, the neurological complications of HSV-2 infection are likely related to a latent reactivation of a previous clinically silent infection, as opposed to one following a primary infection that typically occur in neonates (4). While the fever and urinary tract infection are possibly implicated in the latent viral reactivation in an immune competent state, the precise involved pathways in our patient remain uncertain (13). This case is unique as it underscores the following: (1) Chorea can be among the neurological complications of HSV-2 infection despite normal neuroimaging (14); (2) Although chorea appears to be likely related to active CNS viral infection, the contribution of concurrent CNS autoimmunity affecting basal ganglia cannot be excluded. The rapid resolution of chorea, temporally associated with plasmapheresis rather than an antiviral agent, together with abnormal intrathecal immunoglobulin synthesis and evidenced by elevation of CSF IgG index and detection of oligoclonal bands with CSF restriction in, suggest a potential contributory role of CNS autoimmunity, despite the lack of detectable neuronal autoantibodies in CSF. However, given that serum testing for the neuronal autoantibodies was inadvertently omitted, and that serum testing, compared with CSF testing, can, at times, offer greater sensitivity for detecting certain neuronal autoantibodies, such as Leucine-rich glioma inactivated 1 (LGI-1) autoantibody, one cannot completely exclude the potential contributory role of those neuronal autoantibodies. Further, given the known high and low sensitivity of brain MRI in diagnosing CNS involvement with infection-related encephalitis and inflammation-related autoimmune encephalitis (15–18), respectively, it is conceivable that concurrent CNS autoimmunity, and not active viral infection alone, contributed to CSF lymphocytic pleocytosis. With the lack of detectable neuronal autoantibodies, abnormal MRI findings, and absence of any EEG data or brain biopsy, we may only, at best, fulfill a diagnosis of a possible autoimmune cause of chorea (6). (3) These findings suggest that concurrent autoimmunity might occur in HSV-2 encephalitis. This is in contrast to the commonly observed later-onset immune response associated with sequelae or relapse of CNS HSV-1 infections, which is often associated with neuronal autoantibodies; (4) Recognizing that chorea, among other movement disorders, can be among early presenting symptoms of HSV-2 encephalitis may permit early diagnosis, and adding immune therapies to antiviral agents have the possibility to improve clinical outcomes. However, the optimal time to start immunotherapy has not yet been established. Although, both early and delayed glucocorticoid administration has been shown to equally improve outcomes, it is typically withheld early in the course of active infections in order to presumably limit viral replication (9). In addition, randomized trials examining concurrent administration of glucocorticoid and acyclovir are also inconclusive (19). In this case, early initiation of plasmapheresis clearly yielded full and rapid resolution of chorea. Our case suggests that concurrent immune therapies and antiviral agents might be beneficial in expediting the recovery and limit long-term neurological sequelae.

Finally, a diagnostic challenge lies in that there have been rapidly emerging cases of encephalitis with preceding viral-like illness of suspected immune origin but with undetectable neuronal autoantibodies in serum and CSF and normal neuroimaging as in this case (15–18). This is confounded with an overdependence on antibody status to diagnose encephalitis of suspected immune origin, which may delay diagnosis and treatment (19). Therefore, a high index of clinical suspicion is pivotal to timely diagnose and treat such patients.

## Conclusion

This is the first case associating HSV-2 encephalitis with chorea. The neurological complications, including chorea, are largely related to active CNS HSV-2 infection, possibly together with triggered CNS autoimmunity despite undetectable CSF neuronal autoantibodies and normal neuroimaging. Early diagnosis and treatment with antiviral agents and immune therapies might be pivotal to optimize the clinical outcome.

## Data Availability Statement

The original contributions presented in the study are included in the article/supplementary material, further inquiries can be directed to the corresponding author.

## Ethics Statement

Written informed consent was obtained from the individual(s) for the publication of any potentially identifiable images or data included in this article.

## Author Contributions

MK, AB, NH, and SN contributed to the writing and critical revision of the manuscript. All authors gave important contributions to the final form of the manuscript.

## Conflict of Interest

The authors declare that the research was conducted in the absence of any commercial or financial relationships that could be construed as a potential conflict of interest.

## Publisher's Note

All claims expressed in this article are solely those of the authors and do not necessarily represent those of their affiliated organizations, or those of the publisher, the editors and the reviewers. Any product that may be evaluated in this article, or claim that may be made by its manufacturer, is not guaranteed or endorsed by the publisher.
